# Prognostic value of quantitative and visual electroencephalography in disorders of consciousness: a retrospective study

**DOI:** 10.3389/fnins.2025.1644497

**Published:** 2025-10-14

**Authors:** Yuhei Mori, Kazuko Kanno, Hiroshi Hoshino, Ken Suzutani, Asami Oyama, Shuntaro Itagaki, Yasuto Kunii, Itaru Miura

**Affiliations:** ^1^Department of Neuropsychiatry, School of Medicine, Fukushima Medical University, Fukushima, Japan; ^2^Department of Emergency and Critical Care Medicine, Fukushima Medical University, Fukushima, Japan; ^3^Department of Disaster Psychiatry, International Research Institute of Disaster Science, Tohoku University, Sendai, Japan; ^4^Department of Psychiatry, Graduate School of Medicine, Tohoku University, Sendai, Japan

**Keywords:** brain dysfunction, critical care, electroencephalography, neurology, outcome prediction models

## Abstract

**Background:**

Electroencephalography (EEG) is widely used to assess prognosis in patients with disorders of consciousness (DoC). Visual assessments by physicians and quantitative EEG (qEEG) are commonly used; however, only a few studies have directly compared their predictive accuracy. Therefore, in this study, we aimed to compare the prognostic value of visual EEG classification versus that of qEEG-based spectral analysis for survival and neurological outcomes in patients with impaired consciousness.

**Methods:**

In this retrospective study, we examined 97 patients with impaired consciousness admitted to the Emergency and Critical Care Center of Fukushima Medical University Hospital between April 2018 and December 2023. Visual EEG grading was performed using a conventional grading system based on established criteria. Receiver operating characteristic (ROC) curves were used to compare predictive performance. Multivariate logistic regression models were developed incorporating qEEG and clinical prognostic factors (Scarpino score, rehabilitation status, and age). The incremental predictive value of clinical variables was assessed using DeLong’s test.

**Results:**

Visual EEG assessment showed moderate predictive accuracy [area under the curve (AUC) = 0.77 for survival; 0.677–0.725 for neurological outcomes]. qEEG-based models showed comparable performance to visual EEG classification, with slightly higher AUC values that were not statistically significant. The addition of clinical factors significantly improved predictive accuracy, particularly for neurological recovery (AUC improved from 0.729 to 0.936; *P* < 0.001).

**Conclusion:**

Combining qEEG features and clinical prognostic factors provided a comprehensive approach for outcome prediction in patients with DoC. These findings support the potential of a multimodal prognostic framework integrating objective EEG metrics and physician-derived evaluations, although further prospective validation is required.

## 1 Introduction

Electroencephalography (EEG), an important tool in clinical neurophysiology, is widely used to assess cortical activity and diagnose neurological conditions. Its high temporal resolution enables real-time monitoring of brain activity, making it particularly valuable for critically ill patients with disorders of consciousness (DoC) (Bai et al., 2021; Comanducci et al., 2020). The International Federation of Clinical Neurophysiology and the American Clinical Neurophysiology Society (ACNS) have established standardized guidelines for EEG recording and interpretation, ensuring consistent application, especially in intensive care unit settings (Hirsch et al., 2021).

Traditional visual EEG interpretation involves evaluating several critical parameters, including background rhythm, responsiveness to external stimuli, epileptiform activity, and pathological patterns (burst suppression and electrocerebral inactivity) (Muller et al., 2020; Faro et al., 2019; Synek and Shaw, 1989). These abnormal EEG patterns are strong predictors of poor prognosis in patients with severe brain injuries (Janati, 2011). In particular, epileptiform discharges and burst suppression patterns in post-anoxic encephalopathy have been associated with poor recovery and high mortality rates (Lamartine Monteiro et al., 2016). The five-stage EEG grading system described by Markand (1984), based on earlier frameworks by Hockaday et al. (1965), Prior (1973), Hockaday et al. (1965), is one of the most widely used approaches for assessing EEG abnormalities and their correlation with neurological outcomes. However, visual EEG interpretation is inherently subjective, prone to inter-observer variability, and highly dependent on clinician expertise.

To address these limitations of visual EEG interpretation, quantitative EEG (qEEG) has become a complementary analytical approach. qEEG applies mathematical transformations, including the fast Fourier transform and spectral analysis, to objectively assess EEG signals (Ballanti et al., 2022). This method enables precise measurement of power distribution across frequency bands, improves predictive accuracy, and reduces inter-observer variability. Key qEEG-derived metrics, such as alpha-to-delta power ratios, spectral entropy, and burst suppression ratios, are robust markers of neurological deterioration (Wiley et al., 2018). Furthermore, EEG reactivity tests, which evaluate brainwave responses to external stimuli and evoked potentials, assessing sensory processing, have also proven valuable in predicting neurological outcomes (Pan et al., 2020; Benghanem et al., 2022; Pruvost-Robieux et al., 2022). These quantitative approaches are particularly beneficial in neurocritical care, where precise prognostication guides treatment decisions and facilitates discussions with patients’ families (Curley et al., 2022).

Previous studies have primarily focused on post-anoxic encephalopathy or severe traumatic brain injury (Hofmeijer et al., 2015; Rossetti et al., 2010; O’Donnell et al., 2021), limiting prognostic comparisons between visual EEG assessment and qEEG across diverse etiologies of consciousness disorders. Most existing research has been limited to narrow patient populations, creating a gap in the understanding of EEG-based prognostication in broader clinical settings.

Several EEG-based scoring systems have been proposed for predicting neurological outcomes in patients with DoC, most of which were developed and validated in heterogeneous cohorts predominantly composed of patients with traumatic brain injuries, but also including individuals with anoxic and cerebrovascular etiologies. Estraneo et al. (2016) developed a classification system for diagnostic purposes, categorizing EEG backgrounds by frequency, voltage, and reactivity. However, its design lacked prognostic intent, limiting its predictive utility. Bagnato et al. (2015) subsequently created a prognostic EEG scoring method using the same descriptors (frequency, voltage, and reactivity) and applied it to rehabilitation cohorts, but it lacked comprehensive standardization and integration of multiple EEG parameters. Building upon these frameworks, Scarpino et al. (2019, 2020) applied standardized descriptors from the ACNS classification, including continuity, voltage, frequency, symmetry, and reactivity, to a large cohort of patients with DoC and demonstrated that this approach provided improved prognostic accuracy. Their findings showed that the ACNS-based EEG score outperformed Estraneo’s diagnostic EEG classification and Bagnato’s prognostic EEG score in predicting post-acute neurological recovery. Scarpino’s EEG score achieved the highest area under the curve (AUC) (AUC = 0.79), surpassing Bagnato’s (AUC = 0.71) and Estraneo’s (AUC = 0.70). When combined with the Coma Recovery Scale-Revised score, predictive sensitivity increased to 76%, and specificity reached 93%. These findings show Scarpino’s EEG scoring system to be a significant prognostic tool for DoC.

We hypothesized that qEEG, particularly when combined with clinical scores, would outperform visual EEG in prognostic accuracy. Therefore, in this study, we aimed to primarily compare the prognostic accuracy of visual EEG assessment (EEG grading described by Markand (1984), based on the systems of Hockaday et al. (1965), Prior (1973)) with that of qEEG in patients with DoC, without restricting the cohort to those with post-anoxic encephalopathy or severe traumatic brain injury. Furthermore, we sought to enhance predictive precision by integrating established prognostic factors, including the Scarpino score and other outcome markers, into qEEG analysis. By systematically evaluating the strengths and limitations of both assessment methodologies across a heterogeneous patient cohort, we aim to contribute to the standardization of EEG-based prognostication and enhance clinical decision-making in neurocritical care.

## 2 Materials and methods

### 2.1 Participants

Patients were eligible for inclusion if they met the following criteria: (1) admission to the Emergency and Critical Care Center of Fukushima Medical University between April 2018 and December 2023; (2) impaired consciousness secondary to an identifiable medical condition (cardiac arrest, stroke, infection, metabolic disturbance); and (3) referral to the Department of Neuropsychiatry for EEG examination to evaluate the state of consciousness and neurological prognosis. All patients underwent EEG assessment during the acute or subacute phase of illness following the discontinuation of sedative medications. The Glasgow Coma Scale score at admission was documented to define the level of consciousness. Therapeutic and surgical interventions, including targeted temperature management, mechanical ventilation, and cardiopulmonary resuscitation, were permitted and recorded.

From medical records, the following baseline data were collected: age, sex, date of admission and discharge, primary illness, duration from the onset of impaired consciousness to EEG, GCS score at admission, occurrence of cardiopulmonary arrest, presence of targeted temperature management, rehabilitation interventions, and antiepileptic drug administration status.

Primary etiologies included post-anoxic encephalopathy following cardiac arrest, ischemic and hemorrhagic strokes, metabolic/toxic encephalopathy, and other causes. The distribution by etiology is summarized in Table 1.

**TABLE 1:** Summary of primary illnesses of patients.

Cause	Number	Rate
Acute heart failure due to acute coronary syndrome and cardiomyopathy	13	13%
Acute drug intoxication	2	2%
Acute respiratory distress syndrome due to drowning	2	2%
Anaphylactic shock	2	2%
Arrhythmia	2	2%
Asphyxia	3	3%
Cerebral infarction, hemorrhage	2	2%
Electrolyte abnormality	2	2%
Head injury (–)	25	26%
Heatstroke	1	1%
Hepatic failure	7	7%
Hypoglycemic coma	3	3%
Hypothermia	1	1%
Infection (including septic shock, urinary)	14	14%
Kidney failure	1	1%
Neuroleptic malignant syndrome	1	1%
Rhabdomyolysis	1	1%
Suicide by hanging	4	4%
Unspecified	10	10%
Infection of unknown focus	1	1%

Values are presented as *n* (%) with the % calculated against the total cohort (*n* = 97). “Unspecified” indicates an unclear etiology at discharge after diagnostic work-up. “Infection (unknown focus)” refers to a systemic infection without an identified primary source.

Patients were excluded if they met any of the following criteria: (1) EEG recordings with severe artifacts preventing reliable visual or quantitative analysis; (2) persistent administration of sedative or anesthetic agents at the time of EEG; (3) incomplete clinical or EEG data; or (4) age < 16 years; or (5) EEG not conducted due to logistical limitations; or (6) EEG performed for reasons unrelated to the evaluation of consciousness disturbance. For patients with a history of chronic neurological or psychiatric disorders (epilepsy, dementia), EEGs conducted for reasons other than assessment of impaired consciousness or neurological prognostication were not routinely excluded unless these conditions interfered with the interpretation of EEG or outcome measures. Relevant comorbidities, including cardiovascular and metabolic disorders, were recorded and considered during the statistical analysis. All patients underwent EEG for evaluation of consciousness disturbance and neurological prognosis. The sample size was determined by including all eligible patients who met the inclusion criteria during the study period.

### 2.2 Outcomes

Regarding each prognosis, the prognosis for survival was classified as either survival or death, and neurological outcomes were categorized as recovery to pre-illness state, mild disability, or severe disability. Following the Cerebral Performance Category (CPC) scale, neurological outcomes were defined as follows: CPC1: Recovery to pre-illness state, CPC2: Mild disability, and CPC3–5: Severe disability. If a patient died during hospitalization, the date of death was recorded as the discharge date. In addition, when available, survival status and CPC scores were evaluated at 1 month, 6 months, and 1 year after discharge. The primary endpoint was favorable neurological recovery (CPC1), while survival at discharge served as a secondary endpoint.

### 2.3 EEG acquisition and data processing

Using the international 10–20 system, EEG was recorded from eight scalp electrodes (F3, F4, T3, T4, C3, C4, O1, O2). Power spectra were calculated from a single 10 s artifact-free, eyes-closed resting EEG segment for each patient using fast Fourier transform analysis. In EEG signal processing, short stationary windows of a few seconds are used for spectral estimation (Melman and Victor, 2016; Schomer and Lopes da Silva, 2012). This approach was selected to standardize analysis across heterogeneous recordings and minimize artifact contamination, consistent with prior studies that demonstrate robust EEG markers can be extracted from short stationary segments (Engemann et al., 2018; Sitt et al., 2014), This duration strikes a balance between signal quality and clinical feasibility while aligning with the potential for future automated bedside applications. From the outputs obtained for each electrode, the total power was computed for four frequency bands: δ (1–4 Hz), θ (4–8 Hz), α (8–13 Hz), and β (13–30 Hz). From these data, we derived several functional indices, including occipital alpha (sum of α band outputs from O1 and O2) and average spectral power values across all electrodes (all-average δ, θ, α, or β). Notably, values from the T3 and T4 electrodes were excluded from these calculations. Artifact rejection was performed through visual inspection. Trained EEG technicians and investigators excluded epochs containing eye blinks, muscle activity, electrode pops, or movement artifacts. For quantitative analysis, a 10 s artifact-free resting segment (eyes closed, no stimulation) was selected for each patient. Uniform acquisition filters were applied across all recordings (high-pass time constant 0.1 s ≈ 1.6 Hz, low-pass 60 Hz, notch filter ON) to suppress baseline drift, high-frequency noise, and power-line interference. In this study, EEG was conducted by certified technicians accredited by the Japanese Society of Clinical Neurophysiology. EEG measurements were obtained in the patient rooms of the Emergency and Critical Care Center at our hospital. In all cases, sedative medications were discontinued at the time of examination. The total EEG recording length per patient ranged from 7 to 32 min (median 16 min).

### 2.4 EEG interpretation

Two independent physicians conducted EEG interpretations: one certified by the Japanese Society of Clinical Neurophysiology and the other a board-certified psychiatrist from the Japanese Society of Psychiatry and Neurology with specialized EEG expertise. Both physicians remained blinded to the patients’ clinical backgrounds throughout the evaluation. In addition, the investigators conducting the qEEG analysis were also blinded to the patients’ clinical outcomes. Using the previously described 10 s segment for power spectrum analysis, they graded EEG findings according to the system described by Markand (1984), which is based on the earlier frameworks of Hockaday et al. (1965), Prior (1973). For cases where classification was ambiguous, the final evaluation was determined through consensus between the two physicians. This five-grade system qualitatively assesses the severity of EEG abnormalities, particularly in patients with impaired consciousness. The grading is based on the predominant background activity and its reactivity to external stimuli: Grade 1 (Normal or near-normal): The background consists primarily of alpha activity, occasionally mixed with scattered theta waves. Grade 2 (Mild abnormality): The background is dominated by theta activity, with an admixture of alpha and delta components. Grade 3 (Moderate abnormality): The EEG shows sustained polymorphic delta activity with minimal faster frequencies. The pattern shows variability and retains reactivity to noxious stimulation. Grade 4 (Severe abnormality): The EEG is characterized by predominantly low-amplitude (< 100 μV) delta activity, with no reactivity to any stimuli. Burst suppression patterns may be present. Grade 5 (Profound abnormality): The tracing is nearly flat or shows electrical silence, indicating maximal cortical dysfunction.

Among available EEG scoring systems proposed by Estraneo et al. (2016); Bagnato et al. (2015), Scarpino et al. (2019), the Scarpino score was selected due to its superior prognostic accuracy. Previous comparative analyses have demonstrated that the Scarpino score achieves higher AUC values and improved sensitivity and specificity metrics compared to other systems. EEG recordings were conducted following a referral from the primary emergency care team based on clinical judgment concerning the need for neurological prognostication. As such, the timing of EEG measurements was not standardized across all patients but reflected real-world clinical decision-making in the acute and subacute phases of impaired consciousness. In addition to the Markand grading, the Scarpino EEG score was calculated when sufficient descriptors were available, allowing supplementary comparison of prognostic performance.

### 2.5 Statistical analysis

#### 2.5.1 Clinician-assessed prognostic evaluation

To evaluate the prognostic utility of the visual EEG grading described by Markand (1984), based on Hockaday et al. (1965), Prior (1973), we analyzed two primary outcomes: survival (classified as survival or death) and neurological prognosis [categorized using CPC scores: CPC 1 (recovery to pre-illness state), CPC 2 (mild disability), and CPC 3–5 (severe disability)].

Receiver operating characteristic (ROC) curves were generated to assess predictive accuracy using the pROC package in R (version 3.1.0). Ninety-five percent confidence intervals (Cis) for AUC were estimated using the DeLong method. For neurological prognosis, we employed the one-vs.-all method, creating three separate ROC curve comparisons: Recovery vs. Others (Mild Disability+Severe Disability), Mild Disability vs. Others (Recovery+Severe Disability), and Severe Disability vs. Others (Recovery+Mild Disability). AUC was calculated for each prognosis to evaluate predictive performance. Because group sizes were relatively balanced, no resampling or cross-validation procedures were applied.

In addition to one-vs.-all ROC curves, we performed ordinal logistic regression treating CPC as an ordinal outcome. Model performance was further evaluated with cumulative AUC analysis.

#### 2.5.2 EEG-based prognostic evaluation

Quantitative electroencephalography analysis was conducted using power spectral values from each electrode to predict survival and neurological outcomes. These spectral power values were directly treated as continuous predictor variables. The ground truth labels (survival status and CPC-based neurological categories) were obtained from medical records, independent of EEG evaluation. For each candidate EEG feature, predicted probabilities were generated and ROC curves were constructed by varying classification thresholds. The predicted outcomes were then compared against the ground truth labels to calculate AUC values. Specifically, ROC analyses were conducted using global average powers across all channels in the δ (1–4 Hz), θ (4–8 Hz), α (8–13 Hz), and β (13–30 Hz) bands. In addition, exploratory analyses assessed regional band powers (e.g., occipital α, central β). Parameters with the highest discriminative performance are presented in the Results section.

#### 2.5.3 Univariate analysis for identifying prognostic factors

Univariate analyses were conducted to identify potential prognostic predictors using chi-square tests for categorical variables (cardiopulmonary arrest occurrence, rehabilitation status, photic and nociceptive responses); Mann-Whitney *U*-tests for continuous variables (age, Scarpino score) in binary outcome analyses; and the Kruskal–Wallis test for continuous variables when assessing three-category neurological outcomes.

#### 2.5.4 Multivariate logistic regression analysis

All variables showing significant associations in the univariate analysis (*P* < 0.05) were included in a multivariate logistic regression model to identify independent predictors of survival and neurological prognosis. Adjusted odds ratios (ORs) with 95% CIs were reported.

#### 2.5.5 Multivariate logistic regression incorporating spectral power and clinical predictors

A comprehensive multivariate logistic regression model was constructed incorporating continuous variables (EEG spectral power values and age) and categorical variables (cardiopulmonary arrest occurrence, rehabilitation status, and photic stimulation response) to evaluate electrophysiological and clinical prognostic predictors. Spectral power values were selected based on prior statistical evaluations of survival and neurological outcomes, specifically choosing frequency bands and scalp regions that had shown the highest AUC values in previous univariable analyses. For the final integrated multivariable logistic regression model, we included the spectral feature that demonstrated the strongest univariate association with outcomes, in order to minimize overfitting and to focus on the most robust predictors. Thus, the overall framework for parameter selection was hypothesis-driven, while the final choice of the most predictive spectral feature incorporated a data-driven element. To evaluate whether including clinical predictors could enhance EEG-based model performance, we generated ROC curves for a baseline model (using only spectral power values) and an extended model (integrating additional clinical predictors). The AUC values of these models were computed and compared. Finally, to determine whether the integration of clinical variables significantly improved discriminative performance, we used DeLong’s test for correlated ROC curves. Internal validation was performed using bootstrap resampling with 1,000 iterations. For both the multivariable survival and neurological recovery models, optimism-corrected AUC values were calculated to assess model stability. The bootstrap procedure was implemented using the pROC package in R Statistical Software, and corrected estimates were compared with the original AUCs to evaluate potential overfitting.

**FIGURE 1:**
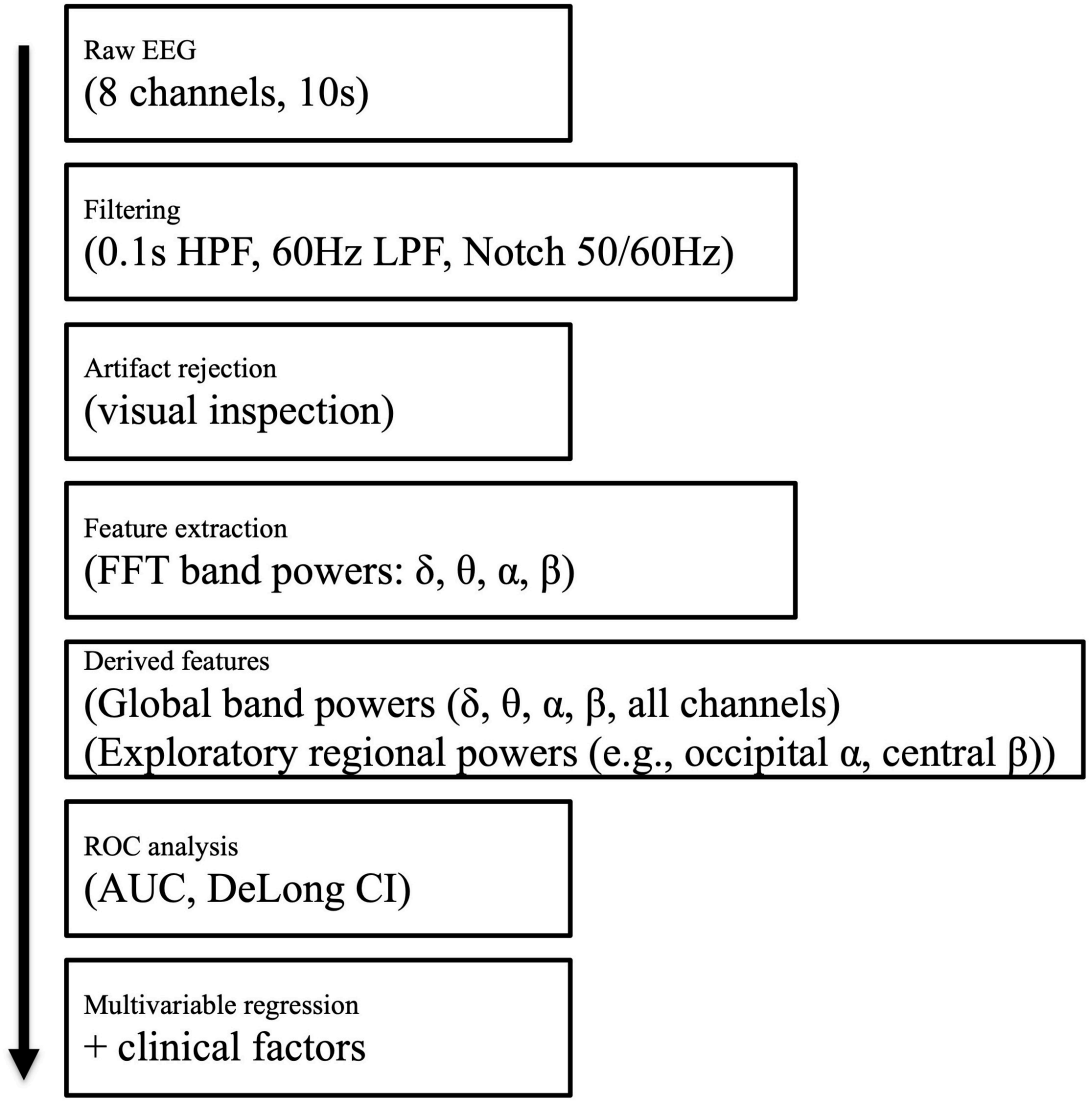
Signal processing workflow for quantitative EEG (qEEG) analysis. Raw EEG (eight channels, 10 s epochs) was subjected to band-pass filtering (0.1–60 Hz) and notch filtering at 50/60 Hz. Artifact-contaminated segments were excluded after visual inspection. Fast Fourier transform (FFT) was applied to extract power spectral densities in the δ (1–4 Hz), θ (4–8 Hz), α (8–13 Hz), and β (13–30 Hz) bands. Global average band powers across all channels were used as the primary qEEG features. In addition, exploratory analyses assessed regional band powers (e.g., occipital α, central β). Receiver operating characteristic (ROC) analysis was performed to calculate the area under the curve (AUC) with 95% confidence intervals (CI) using DeLong’s method. Finally, multivariable regression models were constructed incorporating both qEEG-derived features and clinical variables. HPF, high-pass filter; LPF, low-pass filter.

To improve transparency of the signal processing steps, we provide a schematic flowchart illustrating the preprocessing, feature extraction, and subsequent statistical analyses (Figure 1). This diagram specifies the raw EEG acquisition parameters, filtering procedures, artifact rejection method, extracted quantitative features, and how these features were used to generate ROC curves and multivariable regression models.

#### 2.5.6 Software and computational tools

All statistical analyses were conducted using R Statistical Software (version 3.1.0; Foundation for Statistical Computing). A *P*-value of < 0.05 was considered statistically significant. Bonferroni correction was used to account for multiple comparisons, ensuring the robustness of statistical significance.

### 2.6 Ethics

The Ethics Committee of Fukushima Medical University School of Medicine (REC2023-211) approved this study. It was conducted under the Declaration of Helsinki. As a retrospective medical record review, the study qualified for exemption from individual informed consent requirements. However, we implemented an opt-out process by publicly disclosing study information, ensuring that patients could withdraw if desired.

## 3 Results

### 3.1 Demographic characteristics of study participants

**FIGURE 2:**
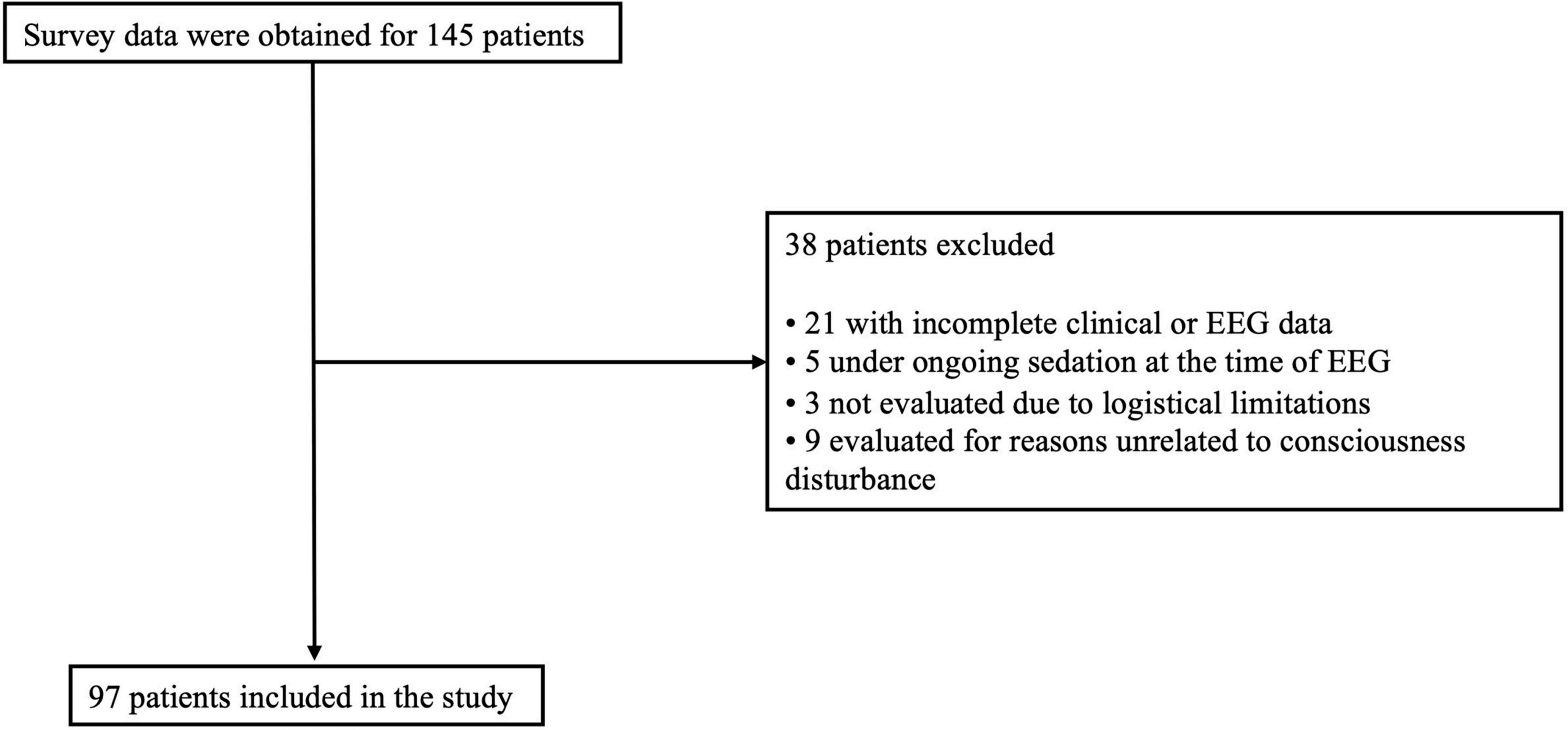
Flow chart describing patient selection. Survey data were obtained for 145 patients. Thirty-eight patients were excluded: 21 owing to incomplete clinical or electroencephalography (EEG) data, five because of ongoing sedation at the time of EEG, three because the EEG was not conducted owing to logistical limitations, and nine because the EEG was conducted for reasons unrelated to the evaluation of consciousness disturbance. Ultimately, 97 patients were included in the final analysis.

A total of 145 patients were initially screened. Of these, 48 were excluded: 21 due to incomplete clinical or EEG data, five because of ongoing sedation at the time of EEG, three not evaluated because of logistical limitations, and nine because EEG was conducted for reasons unrelated to consciousness disturbance. Thus, 97 patients were included in the final analysis (Figure 2 and Supplementary Table 1). The demographic data are summarized in Table 2, and the primary illness distribution is shown in Table 1. For 38 deceased participants, survival duration (in days) from EEG examination to death was recorded.

**TABLE 2:** Demographic characteristics of study participants (*n* = 97).

Variable	Description
Age: mean (SD) (years)	62.6 (18.8)
Sex: male (%)	69 (71)
Hospitalization duration (day): mean (SD)	21.2 (15.8)
Duration between the onset of consciousness impairment and EEG measurement (day): mean (SD)	3.8 (3.1)
Scarpino score	7.1 (2.3)
GCS at admission: mean (SD)	6.2 (4.0)
GCS at EEG measurement: mean (SD)	6.8 (4.1)
GCS at discharge: mean (SD)	7.9 (5.1)
Responsiveness to intermittent photic stimulation at EEG measurement: *n* (%)	10 (10.3)
Responsiveness to pain stimulation response at EEG measurement: *n* (%)	7 (7.2)
Cardiopulmonary arrest (%)	38 (39.2)
Targeted temperature management (%)	36 (37.1)
Rehabilitation intervention (%)	58 (59.8)
Antiepileptic drug administration status: *n* (%)	14 (14.4) [^†^Phenytoin (*n*): 2, Levetiracetam (*n*): 16, Lacosamide (*n*): 2]
**Survival prognosis at the time of discharge: *n* (%)**	
Survival	68 (70.1)
Death	29 (29.9)
Survival prognosis at 1 month following discharge *n* (%)^†⁣†^	24 (24.7)
Survival	20
Death	4
Survival prognosis at 6 months following discharge *n* (%)^†⁣†^	18 (18.6)
Survival	15
Death	3
Survival prognosis at 1 year following discharge *n* (%)^†⁣†^	12 (12.4)
Survival	11
Death	1
**Neurological prognosis at discharge: *n* (%)**	
Positive outcome (CPC 1, 2) (%)	23 (23.7)
Negative outcome (CPC 3, 4, 5) (%)	74 (76.3)
Neurological prognosis at 1 month following discharge: *n* (%)^†⁣†^	21 (21.6)
Positive outcome (CPC 1, 2) (%)	12
Negative outcome (CPC 3, 4, 5) (%)	9
Neurological prognosis at 6 months following discharge: *n* (%)^†⁣†^	16 (16.5)
Positive outcome (CPC 1, 2) (%)	9
Negative outcome (CPC 3, 4, 5) (%)	7
Neurological prognosis at 1 year following discharge: *n* (%)^†⁣†^	10 (10.3)
Positive outcome (CPC 1, 2) (%)	6
Negative outcome (CPC 3, 4, 5) (%)	4

Values are expressed as mean ± SD or *n* (%) as indicated. Days are counted from the onset of impaired consciousness to the indicated event. Follow-up proportions are calculated with the denominator being the total cohort (*n* = 97); the “*n*” shown under each timepoint represents the number of patients successfully followed. GCS, Glasgow Coma Scale; CPC, Cerebral Performance Category; SD, standard deviation.

^†^Antiepileptic drugs among patients receiving AEDs at electroencephalography (EEG): Phenytoin (*n* = 2), Levetiracetam (*n* = 16), and Lacosamide (*n* = 2). Some patients received multiple AEDs. ^††^Number (and % of the total cohort) with available survival/neurological outcomes at each timepoint.

### 3.2 Clinician-assessed prognostic evaluation (visual evaluation)

Overall, 97 patients were evaluated using the five-tier visual EEG grading system described by Markand (1984), which is based on the earlier frameworks of Hockaday et al. (1965), Prior (1973), as assessed by physician evaluation (Table 3). Five patients were excluded owing to EEG findings that could not be classified, specifically because adequate background activity was not present. ROC analysis showed the classification system’s prognostic performance:

**TABLE 3:** Visual electroencephalography (EEG) grading (Markand, 1984) and prognoses at discharge.

Grade	Survival prognosis at discharge		Neurological prognosis at discharge		
	Survival	Death	Recovery	Mild	Severe
Grade 1	12	2	5	4	5
Grade 2	32	5	3	4	30
Grade 3	12	2	4	2	8
Grade 4	0	3	0	0	3
Grade 5	15	6	0	0	21
Burst suppression	2	1	0	0	3
Periodic synchronous discharge	4	1	0	1	4

Numbers are *n* (row totals shown). Survival refers to status at discharge. Neurological outcomes are categorized by CPC: Recovery, CPC 1; Mild, CPC 2; Severe, CPC 3–5. Five EEGs without adequate background activity were not classifiable and are excluded from this table. Burst suppression and periodic synchronous discharge denote pattern-based categories observed outside Grades 1–5. CPC, Cerebral Performance Category.

For survival prognosis (mortality prediction), the AUC was 0.77 (95% CI 0.66–0.89), indicating moderate discrimination between survival and death. For neurological prognosis, the AUC was 0.68 (95% CI 0.52–0.82) for predicting recovery, 0.710 (95% CI 0.55–0.87) for mild disability, and 0.73 (95% CI 0.61–0.83) for severe disability. This grading system showed moderate-to-good predictive accuracy, especially for mild to severe neurological impairment.

### 3.3 EEG-based prognostic evaluation

Table 4 presents the mean spectral power values for each frequency band across electrode sites, along with the corresponding AUC values from ROC analyses for mortality and neurological prognosis. Analysis of spectral power correlations with survival duration revealed a significant positive correlation across all bands. Specifically, the delta band showed a significant positive correlation with survival duration (ρ = 0.346, *P* = 0.033); the theta band showed a stronger significant correlation (ρ = 0.443, *P* = 0.005); the beta band also showed a significant positive correlation (ρ = 0.426, *P* = 0.008); and the alpha band showed the strongest positive correlation with survival duration (ρ = 0.492, *P* = 0.002).

**TABLE 4:** Mean spectral values for each frequency band at each electrode site in the survival and deceased groups, along with AUC values from ROC analysis for predicting mortality and neurological prognosis.

Electrode location	Survivor groups	Non-survivor groups	AUC of the ROC curve for survival prognosis	AUC of the ROC curve for neurological prognosis		
				Recovery	Mild disability	Severe disability
F-delta	60.22	25.94	0.31	0.61	0.62	0.37
F-theta	43.75	9.64	0.74	0.57	0.58	0.61
F-alpha	16.27	2.99	0.80	0.61	0.64	0.36
F-beta	9.071	2.49	0.787	0.68	0.70	0.32
C-delta	51.39	21.97	0.727	0.61	0.61	0.37
C-theta	45.59	9.64	0.757	0.54	0.58	0.58
C-alpha	15.59	3.19	0.815	0.61	0.62	0.37
C-beta	7.26	2.51	0.804	0.70	0.71	0.31
O-delta	31.33	18.08	0.737	0.64	0.58	0.37
O-theta	23.14	9.45	0.75	0.62	0.58	0.60
O-alpha	9.88	2.46	0.79	0.69	0.59	0.33
O-beta	6.88	2.06	0.769	0.726	0.72	0.29
All-average delta†	42.83	18.66	0.704	0.62	0.61	0.37
All-average theta†	32.91	8.19	0.751	0.57	0.58	0.61
All-average alpha†	12.13	2.53	0.804	0.63	0.61	0.36
All-average beta†	7.12	2.17	0.79	0.70	0.76	0.29

“Survivor” and “Non-survivor” columns show the mean spectral power (absolute values in μV^2^/Hz) for each frequency band at the indicated electrode region. The right-hand columns report the area under the ROC curve (AUC) for predicting survival prognosis and neurological outcomes (recovery, mild disability, and severe disability) based on each spectral parameter. AUC (survival), ROC AUC for mortality prediction. AUC (neurological outcomes), one-vs.-rest ROC AUCs for Recovery (CPC 1), Mild disability (CPC 2), and Severe disability (CPC 3–5). F, frontal (F3, F4); C, central (C3, C4); O, occipital (O1, O2). All-average indicates the average across F, C, and O regions. Spectral power was computed from a 10 s, artifact-free, eyes-closed segment per patient using Fast Fourier Transform. Bands were δ (1–4 Hz), θ (4–8 Hz), α (8–13 Hz), and β (13–30 Hz). AUC, area under the ROC curve; ROC, receiver operating characteristic; CPC, Cerebral Performance Category.

^†^Mean value across all electrodes.

### 3.4 Survival and neurological outcome prediction based on physician assessment and qEEG

**FIGURE 3:**
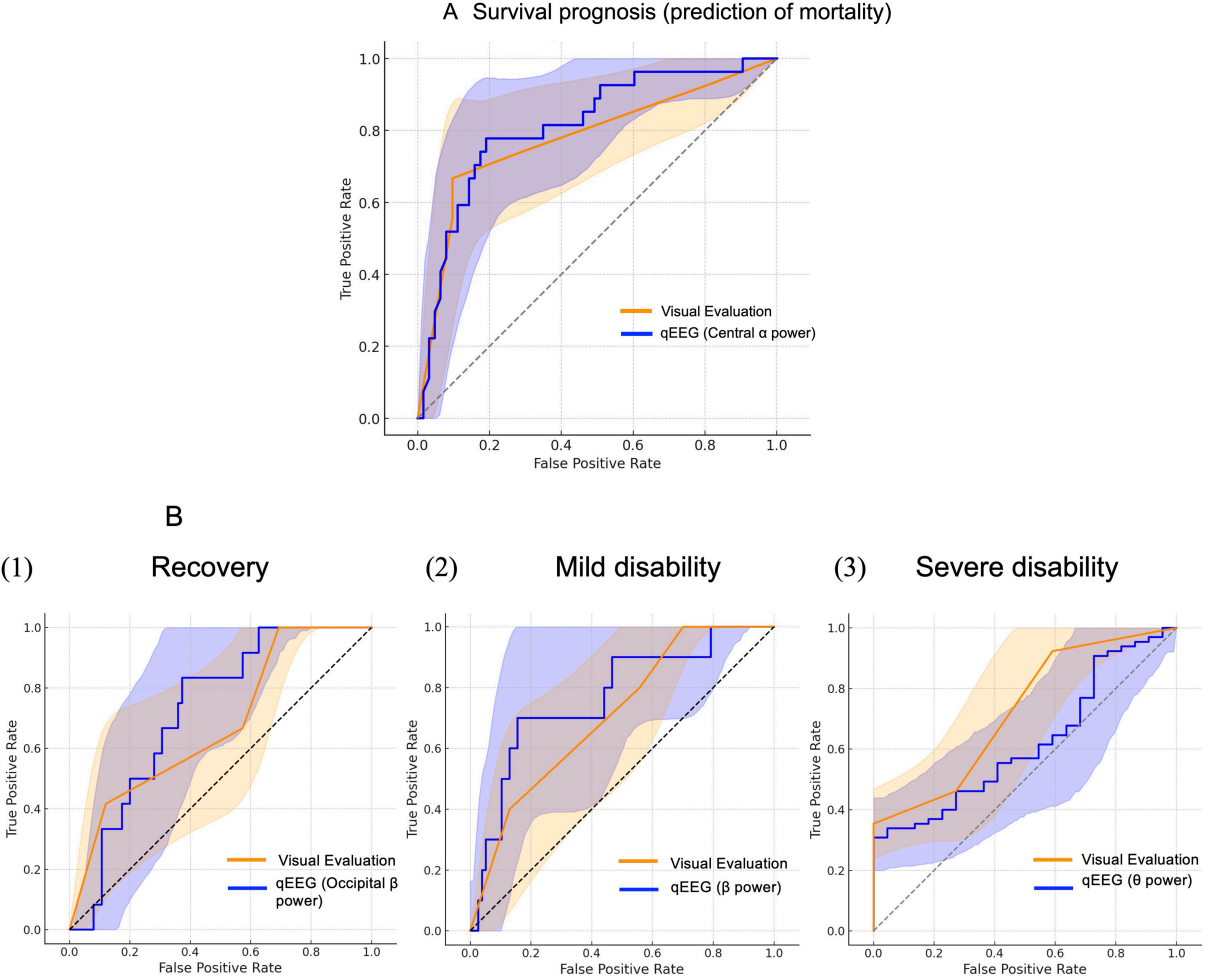
Receiver operating characteristic (ROC) curves for survival and neurological outcome prediction based on physician assessment and quantitative EEG analysis. **(A)** ROC curve for survival prediction: physician vs. quantitative electroencephalography (EEG). ROC curves compare the predictive performance of the physician-based visual EEG grading (Markand, 1984) and quantitative EEG (central alpha power) for mortality prediction. The blue curve represents the Markand grading [area under the curve (AUC) = 0.770], and the red curve represents central alpha power (AUC = 0.815), which showed the highest AUC among all quantitative EEG parameters. The gray dashed line indicates the reference for random prediction. The AUC for central alpha power is higher; however, the difference is not significant (*P* = 0.523, DeLong test). **(B)** ROC curve for neurological outcome prediction: physician vs. quantitative EEG. ROC curves compare the predictive performance of visual EEG grading (blue curves) and quantitative EEG (qEEG; red curves) for neurological outcomes: (1) recovery, (2) mild disability, and (3) severe disability. For each outcome, the qEEG parameter with the highest AUC is selected (occipital β power for recovery, all-average β power for mild disability, and all-average θ power for severe disability; AUCs = 0.729, 0.768, and 0.619, respectively). The gray curves in each outcome represent the Markand grading (AUC = 0.677, 0.710, and 0.725, respectively). None of the differences are significant (*P* = 0.699, 0.621, 0.267; DeLong test).

Figure 3 displays ROC curves that compare physician assessments using the five-tier visual EEG grading system described by Markand (1984), based on Hockaday et al. (1965), Prior (1973), with qEEG parameters for predicting outcomes. ROC curves were generated for each qEEG feature (global and regional band powers), and the parameters with the highest AUC values are presented below as representative findings. For the primary endpoint of favorable neurological recovery (CPC1), the Markand classification yielded an AUC of 0.68 (95% CI 0.52–0.82), which is comparable to the optimal qEEG parameter, occipital beta power, which had an AUC of 0.726 (95% CI 0.59–0.84; DeLong *P* = 0.699). For the secondary endpoint, survival at discharge, the Markand ON classification yielded an AUC of 0.77 (95% CI 0.66–0.89), whereas central alpha power showed the highest AUC among qEEG parameters (0.815, 95% CI 0.71–0.90; DeLong *P* = 0.523). For other neurological outcomes, the Markand classification yielded AUCs of 0.710 for mild disability and 0.73 for severe disability, which were comparable to the corresponding qEEG-based parameters (all-region average beta power for mild disability, AUC = 0.690, 95% CI 0.58–0.92; all-region average theta power for severe disability, AUC = 0.619, 95% CI 0.49–0.75). These models demonstrated moderate to good discriminative performance, particularly for recovery and mild disability. Additionally, DeLong’s test indicated no significant differences between the physician-based and qEEG-based models across all comparisons. Supplementary analyses comparing the Markand and Scarpino systems showed comparable predictive accuracy for survival (Supplementary Table 2).

### 3.5 Univariate analysis for identifying prognostic factors

Factors significantly associated with survival prognosis included cardiopulmonary arrest (chi-square tests, *P* < 0.001), rehabilitation intervention (chi-square tests, *P* < 0.001), responsiveness to intermittent photic stimulation (chi-square tests, *P* = 0.045), and the Scarpino score (Mann–Whitney *U*-test, *P* < 0.001). Conversely, sex (chi-square tests, *P* = 0.468), responsiveness to pain stimulation (chi-square tests, *P* = 0.085), and age (Mann–Whitney *U*-test, *P* = 0.75) showed no significant association. Factors significantly associated with neurological prognosis included rehabilitation intervention (chi-square tests, *P* = 0.046) and responsiveness to intermittent photic stimulation (chi-square tests, *P* < 0.001). Furthermore, age and the Scarpino score were significant factors distinguishing between patients who recovered to their pre-morbid state and those with severe neurological sequelae regarding neurological prognosis (Kruskal–Wallis test, *P* < 0.001). Conversely, cardiopulmonary arrest (chi-square tests, *P* = 0.128), sex (chi-square tests, *P* = 0.139), and responsiveness to pain stimulation (chi-square tests, *P* = 0.147) showed no significant association.

### 3.6 Multivariate logistic regression analysis

#### 3.6.1 Logistic regression to determine neurological outcomes for survival prognosis

A multivariate logistic regression analysis was conducted to examine independent predictors of survival, with survival coded as the dependent variable (1 = survived, 0 = deceased). Predictor variables included CPA status, rehabilitation participation, photic stimulation response, Scarpino score, and age. Results are presented as ORs with 95% CIs. Rehabilitation emerged as the only significant independent predictor (OR = 5.31, 95% CI: 1.85–15.2, *P* = 0.002). Other variables were not statistically significant, including CPA (OR = 0.48, 95% CI: 0.15–1.55, *P* = 0.217), photic response (OR = 1.37, 95% CI: 0.18–9.90, *P* = 0.756), Scarpino score (OR = 1.25, 95% CI: 0.94–1.66, *P* = 0.132), and age (OR = 1.00, 95% CI: 0.97–1.03, *P* = 0.893). The overall model was statistically significant (likelihood ratio test, *P* < 0.001), with Nagelkerke’s pseudo R^2^ of 0.28, indicating moderate explanatory power.

These results show that undergoing rehabilitation is a significant independent predictor of survival, while other clinical factors included in the model did not demonstrate significant associations with survival prognosis.

#### 3.6.2 Logistic regression to determine neurological outcomes for neurological prognosis

An ordinal logistic regression analysis was conducted to examine the association between clinical variables and neurological prognosis, classified into three ordinal categories: severe disability, mild disability, and recovery. Two pairwise comparisons were evaluated: (1) mild disability vs. severe disability and (2) recovery vs. severe disability. The independent variables included CPA, rehabilitation participation, photic stimulation response, Scarpino score, and age.

In the comparison between mild and severe disability, the Scarpino score emerged as a significant predictor (OR = 1.94, 95% CI: 1.07–3.50, *P* = 0.028). Higher Scarpino scores were associated with better neurological outcomes. Other variables, including cardiac arrest (OR = 0.86, 95% CI: 0.20–3.60, *P* = 0.861), rehabilitation (OR = 0.62, 95% CI: 0.15–2.55, *P* = 0.184), photic stimulation response (OR = 1.06, 95% CI: 0.22–5.20, *P* = 0.121), and age (OR = 0.93, 95% CI: 0.87–1.01, *P* = 0.287), showed no significant association with outcome differences. The overall model was statistically significant (likelihood ratio test, *P* = 0.030), with a pseudo R^2^ of 0.21, indicating modest explanatory power.

When comparing recovery to severe disability, the Scarpino score (OR = 3.96, 95% CI: 1.50–10.5, *P* = 0.005) and age (OR = 0.85, 95% CI: 0.77–0.94, *P* = 0.001) showed significant associations. Higher Scarpino scores were associated with increased odds of recovery, while older age was significantly associated with a lower likelihood of neurological recovery. Conversely, cardiac arrest (OR = 0.87, 95% CI: 0.21–3.70, *P* = 0.758), rehabilitation (OR = 0.34, 95% CI: 0.08–1.50, *P* = 0.151), and photic stimulation response (OR = 1.96, 95% CI: 0.35–11.0, *P* = 0.220) showed no significant associations with outcomes. The overall model demonstrated strong explanatory power (likelihood ratio test, *P* < 0.001), with a pseudo R^2^ of 0.63.

Ordinal logistic regression using CPC as an ordinal outcome confirmed that Scarpino score and age were significant predictors of neurological recovery (Supplementary Table 4). Cumulative AUC analysis produced similar results.

These findings show that the Scarpino score is a robust predictor of improved neurological prognosis, showing significance in both pairwise comparisons. Furthermore, advanced age was independently associated with poor recovery. Other clinical variables did not show a significant relationship with neurological outcomes in this analysis.

#### 3.6.3 Multivariate logistic regression incorporating spectral power and clinical predictors

**FIGURE 4:**
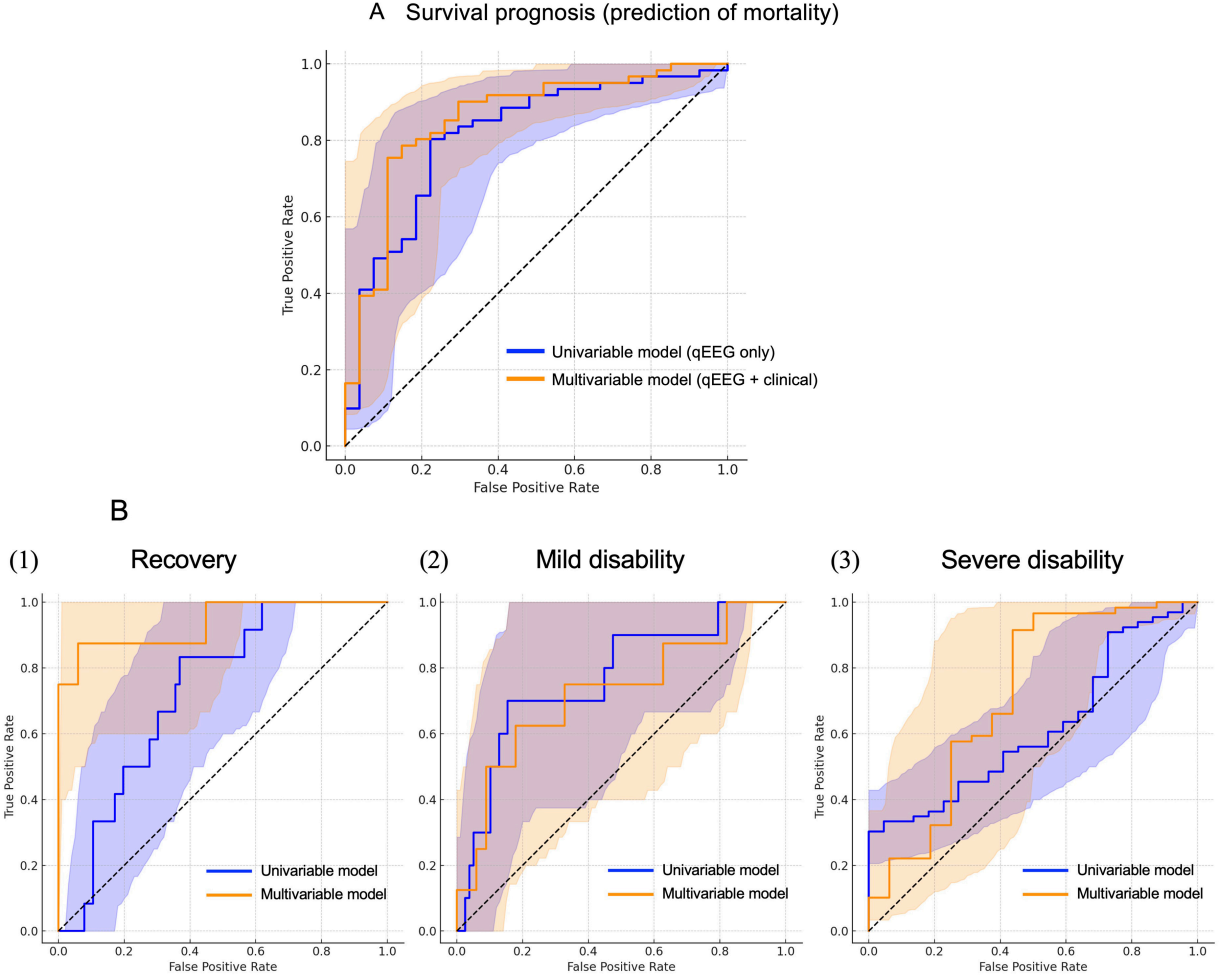
Multivariate logistic regression incorporating spectral power and clinical predictors. **(A)** For survival prognosis, receiver operating characteristic (ROC) curves for life prognosis prediction. The univariable model (blue line) is based solely on parietal alpha power (c_α_power), whereas the multivariable model (orange line) includes additional clinical variables: the presence of cardiopulmonary arrest, rehabilitation status, photic stimulation response, Scarpino score, and age. The multivariable model shows a slightly higher predictive accuracy [area under the curve (AUC) = 0.818] than the univariable model (AUC = 0.815), but the difference is not significant (Z = –0.53, *P* = 0.594; DeLong test). **(B)** For neurological prognosis, ROC curves were generated for predicting neurological outcomes using univariable and multivariable models. A one-vs.-rest approach is applied to each class: (1) recovery, (2) mild disability, and (3) severe disability. The univariable model (quantitative electroencephalography (qEEG) only, blue lines) refers to a logistic regression model that uses only one qEEG power feature (parietal alpha power) as a predictor. The multivariable model (qEEG+clinical, orange lines) includes both qEEG features and additional clinical variables, including cardiopulmonary arrest, rehabilitation status, photic stimulation response, Scarpino score, and age. For recovery, the multivariable model shows substantially better discrimination (AUC = 0.936) than the univariable model (AUC = 0.729), and the difference is significant (Z = 3.94, *P* < 0.001; DeLong test). For mild disability, AUCs are 0.696 and 0.768, respectively, with no significant difference (Z = 1.10, *P* = 0.272). For severe disability, the multivariable model achieves an AUC of 0.854, versus 0.614 for the univariable model; the difference shows a trend but did not reach statistical significance (Z = 1.71, *P* = 0.088).

For survival prognosis, ROC curves were constructed to compare the predictive performance of univariable and multivariable models for outcomes (Figure 3). The multivariable model yielded an AUC of 0.818 (95% CI 0.76–0.94), slightly higher than the AUC of the univariable model (0.815). This difference was not significant (*P* = 0.594; DeLong test), indicating that the inclusion of additional clinical variables did not improve predictive performance. For neurological prognosis, ROC curves were generated similarly to evaluate the predictive performance of univariable and multivariable models (Figure 4). A one-vs-rest approach was applied to each class: recovery, mild disability, and severe disability. For recovery, the multivariable model showed substantially better discrimination (AUC = 0.936) (95% CI 0.81–0.98) compared with the univariable model (AUC = 0.726), and the difference was significant (*P* < 0.001; DeLong test). For mild disability, AUCs were 0.696 and 0.768, respectively, with no significant difference (*P* = 0.272). For severe disability, the multivariable model achieved an AUC of 0.854 versus 0.619 for the univariable model; the difference showed a trend but did not reach statistical significance (*P* = 0.088). Internal validation using bootstrap resampling (1,000 iterations) yielded optimism-corrected AUCs that were nearly identical to the original estimates for both survival and recovery models (Supplementary Table 3).

## 4 Discussion

In this study, we investigated the comparative prognostic value of visual EEG assessment and qEEG analysis in patients with DoC while integrating clinical prognostic factors to develop a multimodal prediction model. Unlike many previous studies that focused primarily on post-anoxic encephalopathy or severe traumatic brain injury, our cohort encompassed a broader spectrum of etiologies. This enabled a more comprehensive evaluation of EEG-based prognostication across heterogeneous clinical contexts. Our findings, consistent with recent literature, underline the nuanced but additive role qEEG plays when combined with traditional clinical and visual EEG metrics.

### 4.1 Visual EEG vs. qEEG: comparative prognostic value

Visual EEG interpretation using the EEG grading described by Markand (1984) showed moderate predictive accuracy for survival (AUC = 0.770) and varying degrees of neurological outcomes. While qEEG features, particularly central alpha power, demonstrated a marginally higher AUC (0.815), this difference was not significant (*P* = 0.523). These findings align with the results of previous studies, indicating that while visual EEG remains useful in acute and subacute stages (Muller et al., 2020), qEEG offers complementary objectivity and reproducibility (Ballanti et al., 2022; Curley et al., 2022). Notably, the ability of central alpha power to achieve comparable predictive performance (AUC = 0.815) to that of expert-rated visual EEG (AUC = 0.770) may hold important clinical implications. Although the difference was not significant, qEEG’s ability to match expert human interpretation suggests potential utility. Unlike visual EEG, which requires specialized training and manual review, qEEG provides objective, reproducible results efficiently. This makes it potentially useful as an adjunct in high-volume clinical settings or institutions lacking EEG specialists. Nevertheless, qEEG offers several important advantages: it provides objective and reproducible quantification less dependent on individual expertise, captures subtle spectral changes not readily discernible by visual inspection, and enables integration with multivariate and machine-learning approaches for prognostication. Future research incorporating non-linear and connectivity-based qEEG metrics may further enhance prognostic sensitivity and complement traditional visual EEG interpretation.

### 4.2 Spectral features and their relationship to survival

Central alpha, theta, and beta band powers showed significant positive correlations with survival duration (all *P* < 0.01), reinforcing prior findings that preserved high-frequency EEG rhythms reflect intact cortico-thalamic function and better prognosis (Simis et al., 2020; Zhou et al., 2023). In contrast, delta activity showed only a weak correlation with survival, likely reflecting a nonspecific marker of severe consciousness impairment (Bagnato et al., 2015). These findings align with current network-based models of consciousness (Babiloni et al., 2016).

### 4.3 Prognostic value of alpha and beta power in DoC

Our analysis shows central alpha power as the most reliable survival predictor, with global alpha and beta power correlating significantly with survival duration. These findings align with established models of cortical-thalamic network dynamics and hypoxic-ischemic pathophysiology. The Global Neuronal Workspace model and Integrated Information Theory explain the essential role of thalamocortical integration in maintaining consciousness, where alpha oscillations, primarily generated using thalamocortical loops, serve as key mediators of network coherence (Mashour et al., 2020; Tononi et al., 2016). Central alpha power reflects the integrity of this network, explaining its strong association with survival outcomes. The balance of thalamocortical input strength sustains spectral transmission, and disruptions in this balance impair cortical oscillations, leading to reduced alpha power and compromised survival (Saponati et al., 2022; Dickey et al., 2024). Hypoxic-ischemic injury induces NMDA receptor-mediated excitotoxicity, causing calcium influx and oxidative stress that preferentially affect thalamic and cortical neurons, disrupting thalamocortical synchrony and reducing alpha power, which explains its prognostic relevance (Zhang et al., 2020; Li and Wang, 2016). Furthermore, the disruption of these networks is associated with poor outcomes in DoC, as the restoration of cortico-thalamic connectivity is essential for functional recovery (Panda et al., 2022; Edlow et al., 2021). The observed association of global beta power with survival duration suggests that excitotoxic damage to cortical circuits, which diminishes the generation of high-frequency beta oscillations, may further contribute to adverse outcomes. Given these findings, qEEG parameters, particularly alpha and beta power, offer an objective and reproducible framework for prognostication in DoC.

### 4.4 Multimodal prediction and integration of clinical features

In this study, rehabilitation interventions were significantly associated with improved survival outcomes in patients with DoC, reinforcing evidence of early, intensive rehabilitation. For example, Zhang et al. (2023) demonstrated that patients receiving specialized acute-phase rehabilitation had significantly higher consciousness recovery rates and medical independence, reducing fatal complications such as aspiration pneumonia. Similarly, a long-term follow-up study by Katz et al. (2014) reported that many minimally conscious patients eventually regained household independence or returned to work or school, with early rehabilitation and consciousness improvement as key prognostic factors.

Our findings extend these previous observations by incorporating a multimodal predictive model for mortality in patients with DoC. Central alpha power, measured via qEEG, emerged as a strong independent prognostic marker. Including multiple clinical features in a multivariable model did not substantially enhance predictive performance (AUC). Cardiopulmonary arrest, photic stimulation response, Scarpino score, and age were significant in univariate analyses; however, only rehabilitation intervention remained an independent predictor in the final model. This highlights the particularly strong impact of rehabilitation on survival beyond other risk factors. Taken together, these results underscore the dual importance of physiological biomarkers, specifically central alpha power, and timely rehabilitation for predicting and improving survival in patients with DoC. However, the limited sample size may have obscured subtle effects from other clinical variables, warranting larger-scale studies.

Scarpino score, age, and EEG reactivity were significant independent predictors in multivariable models. Combining these with qEEG features significantly improved neurological recovery prediction (AUC increased from 0.729 to 0.936, *P* < 0.001), supporting prior literature on clinical-electrophysiological synergy (Toppi et al., 2024; Liu et al., 2024; Bauerschmidt et al., 2021). Our findings show the synergistic effect of integrating clinical variables with qEEG, resulting in a predictive model that significantly surpasses the accuracy of previous approaches. Unlike prior studies that primarily focused on predicting mortality or broad neurological outcomes, our model specifically targets the prediction of favorable neurological recovery (CPC 1), achieving a high AUC of 0.936 in this cohort (*P* < 0.001). Earlier models by Scarpino et al. (2020); Estraneo et al. (2016), Bagnato et al. (2015) targeted different endpoints using distinct methodologies. For instance, Scarpino et al. (2020) used an EEG scoring system based on the ACNS classification, combined with CPC evaluation, to predict mortality and long-term neurological outcomes, achieving an AUC of 0.79. Estraneo et al. (2016) used a standard EEG classification model with an emphasis on predicting the recovery of consciousness from unresponsive wakefulness syndrome to a minimally conscious state, with an AUC of 0.70. Similarly, Bagnato et al. (2015) used a model based on EEG reactivity, voltage, and frequency to predict transitions from UWS to MCS, reporting an AUC of 0.71. In contrast, our study integrated qEEG-derived quantitative features, clinical factors, and EEG reactivity into a unified multimodal framework, which enhanced the prediction of favorable neurological recovery (CPC 1). However, direct comparisons between studies should be interpreted with caution due to methodological differences. By focusing on this specific and clinically significant outcome, our model demonstrated high predictive precision and may provide a more nuanced and comprehensive prognostic approach compared to earlier models. Furthermore, this high-precision model has the potential to serve as a decision-support tool, assisting in family counseling and treatment stratification for patients with DoC. The objective and reproducible nature of our approach may facilitate more personalized and informed decision-making between clinicians and families, but prospective validation is required before its clinical utility can be established. Notably, this significant improvement was observed primarily in the recovery group (CPC 1) but not in mild/severe disability cases. Recovering patients may retain partially preserved cortico-thalamic networks and residual neuroplasticity, whereas severe disability (CPC 4–5) likely reflects irreversible damage, limiting qEEG’s discriminative power. Furthermore, visual EEG often misses covert awareness or microstate shifts detectable by qEEG, making it particularly useful in distinguishing recovering patients (Li et al., 2025; Forgacs et al., 2014). Notably, mild disability prediction did not improve in the multivariable model and was slightly lower compared with the qEEG-only model. This may reflect the intrinsic ambiguity of mild disability as a prognostic category, lying between clear recovery and severe impairment. Clinical variables may not sufficiently differentiate this intermediate state, and their inclusion could have introduced noise that reduced model discriminability. Furthermore, the limited sample size in the mild disability group and potential overlap with adjacent outcome categories may have contributed to the reduced AUC.

From a statistical standpoint, binary outcome modeling (recovery vs. non-recovery) provides clearer discrimination than multi-class stratification (mild vs. severe), explaining the sharper AUC increase for recovery prediction. Finally, recovery outcomes are more strongly associated with modifiable clinical factors, such as rehabilitation and age, whose synergistic effects with EEG metrics further enhance model performance (Liu et al., 2024).

### 4.5 Emerging EEG approaches beyond spectral analysis

Recent large-scale studies have employed machine learning approaches and nonlinear EEG features for outcome prediction in DoC (Sitt et al., 2014; Panda et al., 2022). These approaches, including entropy, functional connectivity (phase lag index), and network dynamics, have demonstrated promising performance and may complement conventional spectral power analysis. Beyond spectral features, additional EEG metrics such as microstates and event-related potentials (e.g., P300, mismatch negativity) also offer prognostic value (Kotchoubey, 2017). Nonlinear features show promise in identifying latent signs of network reorganization and covert awareness (Kulyk, 2019). Furthermore, machine learning models that incorporate multiple qEEG features achieve higher classification accuracy and may reduce diagnostic uncertainty (Stefan et al., 2018). Conventional visual EEG analysis is often limited by brief recording periods. Prolonged EEG captures diurnal patterns and sleep spindles, features associated with cortico-thalamic integrity and improved recovery rates (Forgacs et al., 2014; Grigg-Damberger et al., 2022; Malinowska et al., 2013). Spindles, K-complexes, and cyclic alternating patterns represent key markers for incorporation into future qEEG prognostic frameworks.

### 4.6 Limitations

This study has several limitations. First, its retrospective, single-center design limits generalizability. Second, EEG referral and timing were clinician-driven and not standardized, which may have introduced selection bias. Third, artifact rejection relied on visual inspection rather than automated approaches (e.g., ICA), potentially limiting reproducibility. However, all recordings were processed under identical acquisition filters, and artifact-free segments were consistently selected, ensuring comparability across patients. Future studies may benefit from incorporating automated artifact removal methods to enhance reproducibility. Fourth, the brief EEG recordings (10 s epochs) may have missed important features, such as reactivity and sleep elements. Moreover, since only one artifact-free 10 s segment was chosen for each patient, the representativeness of this selected epoch may be limited. This selection process could introduce bias; thus, future studies should consider using multiple or longer EEG segments to enhance robustness. Fifth, five patients were excluded due to inadequate background activity. This may have selectively removed the most severe cases, potentially leading to an overestimation of prognostic performance. Sixth, our multivariate models included several predictors relative to the sample size, which may increase the risk of overfitting. Although we restricted the number of predictors to those significant in univariate analyses, we additionally performed bootstrap validation, which confirmed the stability of our findings. Nonetheless, external validation in independent cohorts remains necessary. Seventh, although CPC is an ordinal outcome, we used a one-vs.-all ROC approach for comparability with previous studies. This approach does not fully capture the ordinal structure of CPC, and future studies with larger cohorts should consider ordinal logistic regression or cumulative AUC methods. Eighth, the exclusion of patients with incomplete EEG or clinical data may have introduced selection bias. Ninth, our analysis focused solely on conventional spectral power features, omitting non-linear or connectivity-based metrics, such as entropy and complexity. Including these measures in future studies could yield complementary and potentially more sensitive prognostic information. Finally, the visual EEG interpretation in this study relied exclusively on the EEG grading described by Markand, which may limit generalizability. While this EEG grading is widely used for encephalopathy prognosis and served as the basis for our main visual EEG analysis, supplementary analyses using the Scarpino system yielded comparable results, thereby partially supporting the generalizability of our findings. Future studies should compare multiple validated systems, such as ACNS grading and the Synek classification, to further enhance external validity. Future prospective, multicenter studies using long-term recordings and multimodal EEG features are warranted. Our findings show that even short-duration, bedside EEG recordings may provide clinically relevant prognostic information when integrated with structured clinical assessments. Incorporating simplified qEEG metrics into routine protocols could enhance the training of non-specialist clinicians and support timely decision-making in neurocritical care settings. As this was a retrospective study, the precise timing of EEG recordings following the discontinuation of sedative medications was not consistently documented and, therefore, could not be analyzed. This represents a methodological limitation that may have influenced the interpretation of EEG results. Additionally, the Coma Recovery Scale-Revised, which is considered the gold standard for assessing DoC, was not routinely administered in our cohort. Consequently, we were unable to determine patients’ levels of consciousness using Coma Recovery Scale-Revised scores.

## 5 Conclusion

In conclusion, our findings suggest that combining qEEG features with clinical prognostic factors may improve outcome prediction in DoC. However, due to the retrospective design and the short duration of the EEG recordings, these results should be considered preliminary. Future prospective studies utilizing longer and standardized EEG protocols are necessary to validate the clinical utility of qEEG as a bedside decision-support tool.

## Data Availability

The data analyzed in this study is subject to the following licenses/restrictions: not applicable. Requests to access these datasets should be directed to yuhei-m@fmu.ac.jp.
