# Supplementary material for: Prognostic value of quantitative and visual electroencephalography in disorders of consciousness: a retrospective study
Source: Front Neurosci. 2025 Oct 14;19:1644497. doi: 10.3389/fnins.2025.1644497 (PMC12558952; doi:10.3389/fnins.2025.1644497)
Supplement: Supplementary file 1 [file Table_1.docx]

Supplementary Material

**Supplementary Table S1. Reasons for exclusion due to incomplete EEG or clinical data (n = 21).**

| **Reason for exclusion** | **n** | **Details of missing data** |
| --- | --- | --- |
| Incomplete EEG recording | 6 | Recording interrupted due to technical issues or insufficient duration for quantitative analysis |
| Severe artifacts | 5 | Persistent movement/EMG artifacts hindered reliable interpretation, and no 10-second artifact-free epoch could be selected |
| Missing clinical outcome data | 4 | CPC score at discharge not documented in the medical record |
| Loss of EEG data files | 3 | Data corruption or storage error prevented the retrieval of EEG files for analysis |
| Incomplete follow-up | 3 | Survival status available, but CPC scores at discharge or follow-up not recorded |

CPC: Cerebral Performance Category, EEG: Electroencephalography, EMG: Electromyography,

## Supplementary Table S2. Comparison of Visual EEG Systems

| Visual EEG system | AUC (95% CI) for survival prediction |
| --- | --- |
| Markand grading system | 0.77 (0.66–0.89) |
| Scarpino EEG score | 0.81 (0.70–0.90) |

## Supplementary Table S3. Internal Validation Using Bootstrap Resampling

| Model | Original AUC (95% CI) | Bootstrap-corrected AUC | Optimism |
| --- | --- | --- | --- |
| Survival (multivariable) | 0.818 (0.76–0.94) | 0.812 | 0.006 |
| Recovery (multivariable) | 0.936 (0.81–0.98) | 0.928 | 0.008 |

## Supplementary Table S4. Ordinal Logistic Regression for CPC Outcomes

| Predictor | OR (95% CI) | p-value |
| --- | --- | --- |
| Scarpino score | 1.72 (1.20–2.47) | 0.003 |
| Age | 0.89 (0.82–0.96) | 0.002 |
| CPA | 0.91 (0.35–2.34) | 0.84 |
| Rehabilitation | 1.45 (0.58–3.61) | 0.42 |
| Photic response | 1.68 (0.54–5.23) | 0.37 |
